# Urodynamics for Prostate Surgery Trial; Randomised Evaluation of Assessment Methods (UPSTREAM) for diagnosis and management of bladder outlet obstruction in men: study protocol for a randomised controlled trial

**DOI:** 10.1186/s13063-015-1087-1

**Published:** 2015-12-10

**Authors:** K. Bailey, P. Abrams, P. S. Blair, C. Chapple, C. Glazener, J. Horwood, J. A. Lane, J. McGrath, S. Noble, R. Pickard, G. Taylor, G. J. Young, M. J. Drake, A. L. Lewis

**Affiliations:** School of Social and Community Medicine, University of Bristol, Canynge Hall, 39 Whatley Road, Bristol, BS8 2PS UK; Bristol Randomised Trials Collaboration (BRTC), University of Bristol, Canynge Hall, 39 Whatley Road, Bristol, BS8 2PS UK; North Bristol NHS Trust, Bristol Urological Institute, Level 3, Learning and Research Building, Southmead Hospital, Bristol, BS10 5N UK; Sheffield Teaching Hospitals NHS Trust, Room H26, H-Floor, Royal Hallamshire Hospital, Glossop Road, Sheffield, S10 2JF UK; Health Services Research Unit, University of Aberdeen, 3rd Floor, Health Sciences Building, Foresterhill, Aberdeen, AB25 2ZD Scotland; Exeter Surgical Health Services Research Unit – Urology, Royal Devon and Exeter Hospital, Barrack Road, Exeter, Devon EX2 5DW UK; Institute of Cellular Medicine, University of Newcastle, 3rd Floor, William Leech Building, Newcastle upon Tyne, NE2 4HH UK; University of Plymouth, Plymouth, Devon PL4 8AA UK; School of Clinical Sciences, University of Bristol, 69 St Michael’s Hill, BS2 8DZ Bristol, UK; Bristol Randomised Trials Collaboration, University of Bristol, St. Michael’s Hospital, Level D, Southwell Street, Bristol, UK

**Keywords:** UPSTREAM, Urodynamics, Prostate, Surgery, Lower urinary tract symptoms, Bladder outlet obstruction, Benign prostatic obstruction, Randomised controlled trial

## Abstract

**Background:**

Lower urinary tract symptoms (LUTS) comprise storage symptoms, voiding symptoms and post-voiding symptoms. Prevalence and severity of LUTS increase with age and the progressive increase in the aged population group has emphasised the importance to our society of appropriate and effective management of male LUTS. Identification of causal mechanisms is needed to optimise treatment and uroflowmetry is the simplest non-invasive test of voiding function. Invasive urodynamics can evaluate storage function and voiding function; however, there is currently insufficient evidence to support urodynamics becoming part of routine practice in the clinical evaluation of male LUTS.

**Design:**

A 2-arm trial, set in urology departments of at least 26 National Health Service (NHS) hospitals in the United Kingdom (UK), randomising men with bothersome LUTS for whom surgeons would consider offering surgery, between a care pathway based on urodynamic tests with invasive multichannel cystometry and a care pathway based on non-invasive routine tests. The aim of the trial is to determine whether a care pathway not including invasive urodynamics is no worse for men in terms of symptom outcome than one in which it is included, at 18 months after randomisation. This primary clinical outcome will be measured with the International Prostate Symptom Score (IPSS). We will also establish whether inclusion of invasive urodynamics reduces rates of bladder outlet surgery as a main secondary outcome.

**Discussion:**

The general population has an increased life-expectancy and, as men get older, their prostates enlarge and potentially cause benign prostatic obstruction (BPO) which often requires surgery. Furthermore, voiding symptoms become increasingly prevalent, some of which may not be due to BPO. Therefore, as the population ages, more operations will be considered to relieve BPO, some of which may not actually be appropriate. Hence, there is sustained interest in the diagnostic pathway and this trial could improve the chances of an accurate diagnosis and reduce overall numbers of surgical interventions for BPO in the NHS. The morbidity, and therapy costs, of testing must be weighed against the cost saving of surgery reduction.

**Trial registration:**

Controlled-trials.com - ISRCTN56164274 (confirmed registration: 8 April 2014).

**Electronic supplementary material:**

The online version of this article (doi:10.1186/s13063-015-1087-1) contains supplementary material, which is available to authorized users.

## Background

The protocol for this study, Urodynamics for Prostate Surgery Trial; Randomised Evaluation of Assessment Methods (UPSTREAM) for diagnosis and management of bladder outlet obstruction (BOO) in men, describes a major multicentre United Kingdom (UK) trial to establish whether a care pathway not including invasive urodynamics is no worse than one in which it is included in men who are considering further treatment where surgery might be an option for BOO. The study is designed to be as informative as possible, whilst remaining simple and pragmatic, both for those participating and for those involved in clinical care. Research nurses and urologists in each centre will identify and recruit men who are seeking further treatment, which might include surgery, for BOO and collect descriptive information, symptom assessment, flow rates and urinalysis. Those who are eligible will be invited to enter a randomised trial whereby treatment is based on two different diagnostic pathways; either routine information only (the ‘non-urodynamic assessment’ control group) or routine information supplemented by urodynamic testing. All men will be followed up at 6, 12 and 18 months after randomisation.

Lower urinary tract symptoms (LUTS) comprise storage symptoms (e.g. increased daytime urinary frequency, nocturia, urgency, incontinence), voiding symptoms (e.g. slow stream, intermittency, hesitancy, straining, dribbling) and post-voiding symptoms (e.g. post-micturition dribble). Ninety percent of men aged 50 to 80 years suffer from at least 1 LUTS, which can affect quality of life, occupation and other activities. Prevalence and severity increase with age [1] and the progressive increase in the aged population group has emphasised the importance to our society of appropriate and effective management of male LUTS.

Identification of causal mechanisms is needed to optimise treatment. In men with voiding LUTS, benign prostate enlargement (BPE) with ageing causes partial BOO, a situation known as benign prostatic obstruction (BPO). BPO is a major contributor to LUTS. For such patients, prostate surgery, such as transurethral resection of the prostate (TURP), has a good chance of improving LUTS. However, voiding LUTS can also be caused by bladder dysfunction: e.g. poor expulsion strength of the bladder muscle. This is called ‘underactive bladder, or ‘detrusor underactivity’, as the main bladder muscle anatomically is called the detrusor. In such men, it is hard to justify prostate surgery if BPO is not present, especially in view of potential adverse effects associated with surgery, such as blood transfusion requirement, problems of sexual function, anaesthetic problems or incontinence.

Tests of lower urinary tract function are used in clinical practice to demonstrate the causes of voiding or storage problems. Symptom scores are recorded and a physical examination is performed followed by urinalysis. Uroflowmetry is the simplest non-invasive test of voiding function. It entails voiding into a recording device that measures the volume of urine passed and the rate of urine flow, with an ultrasound scan after voiding to see how efficiently the bladder has emptied. In addition, the National Institute for Health and Care Excellence (NICE) guidance on management of LUTS in men (CG97) [2], states that invasive urodynamics may be used when invasive treatment is being considered, or for equivocal or more complex cases. Invasive urodynamics, which is also called multichannel cystometry, employs bladder catheterisation for both bladder filling and bladder pressure measurement, and rectal catheterisation for measurement of abdominal pressure. Concurrent subtraction of abdominal from bladder pressure by a computer calculates ‘detrusor pressure’, to demonstrate whether bladder contraction is occurring. Thus, invasive urodynamics can evaluate storage function (while the bladder is being filled) and voiding function (when the man passes urine). Observing high detrusor pressure associated with only a low urine flow rate is diagnostic of BOO [3]. Low detrusor pressure with low flow implies that bladder contractility is impaired [4]. Without invasive urodynamics, it is uncertain for any given individual whether the bladder outlet is obstructed, and whether the bladder is overactive during storage or underactive during voiding.

Other diagnostic methods have been evaluated and reported with up to level-3 evidence. These include: penile cuff test [5, 6]; urethral reflectography [7]; ultrasound measurement of bladder wall thickness and weight [8]; intravesical prostatic protrusion [9]; resistive index [10]; and prostatic urethral angle [11]. However, there is insufficient evidence to warrant any of these tests becoming standard practice in the clinical evaluation of male LUTS [12].

### Health Technology Assessment (HTA) programme

Despite the implicit merit of confirming that BOO is present before proceeding to surgery to relieve BOO, the lack of relevant research evidence means that many centres omit the test from the usual care diagnostic pathway. Invasive testing is perceived as unpleasant and service delivery has cost implications. NICE CG97 indicates that performing an invasive procedure is a balance of the possible benefits versus the possible risks, and that these must be explained to the patient during informed consent for the procedure, and appropriate advice given regarding potential adverse events.

The HTA addressed this with a commissioning brief asking the research question: In men considering surgery for bothersome LUTS, is diagnostic categorisation using results of invasive multichannel urodynamics worthwhile from the perspective of the men concerned and the National Health Service (NHS) compared to not using multichannel urodynamics?

This research was commissioned by the NIHR-HTA following prioritisation of research questions posed by the NICE Guideline Development Group for male LUTS, which indicated that research into the role of invasive urodynamics would clarify whether it could improve the outcome of surgery, and whether it should be recommended or not in the future [13]. They considered that improving the chance of an accurate diagnosis and identifying potential complications was the most important outcome when considering surgical treatment.

### Population

Forty-four thousand new cases of symptomatic BPO are diagnosed each year. Since BPO is a disease of older men [14], the number of patients affected is likely to increase by almost 50 % by the year 2025, in line with population ageing.

Disease-specific health-related quality of life (HRQOL) measures are significantly worse in men with higher symptom severity ratings in population-based studies [15]. Severe LUTS may require surgical treatment, and 25,000 surgical procedures to relieve BPO are currently performed each year in the NHS. The most widely-used approach is TURP using monopolar or bipolar electrodes, or less commonly laser ablation [16].

### Rationale for current study

The NICE clinical guideline group on male LUTS [2] was unable to identify any methodologically high-quality clinical or economic studies. The literature has been reviewed by Parsons and colleagues [12] and by various professional groups in the last decade: most recently the Cochrane Database Systematic Review, Invasive urodynamic studies (UDS) for the management of LUTS in men with voiding dysfunction [17]. None of these reviews was able to identify high-level evidence on the use of invasive urodynamic testing in male LUTS.

We reviewed evidence for the diagnostic role of invasive urodynamics in men with LUTS prior to surgery for BPO. We identified no published randomised controlled trials (RCTs) with data comparing the standard practice investigation [18] (urine flow rate measurement (maximum urinary flow rate (Q_max_)) and ultrasound estimate of post-void residual urine (PVR)), with invasive urodynamics. One abstract did not have any useable data [19].

Level-3 evidence exists to suggest that patient selection after invasive urodynamics maximises the outcome benefits to patients from surgery to relieve BPO, over and above that given by the standard investigations of Q_max_ and PVR. The American Urological Association (AUA) guidelines recommend that the greater diagnostic benefits of invasive urodynamics over Q_max_/PVR are discussed with patients prior to the decision for prostate surgery [20].

From NHS reference costs data, 98,986 UDS were undertaken on men and women by 131 NHS Trusts in England in 2011–2012 at a tariff cost of £16.7 million; no information for use of invasive urodynamics specifically in men is available.

NHS Health Episode Statistics show that approximately 25,000 TURPs are performed annually. For 100 procedures, the specific equipment and consumables cost of TURP is approximately £29,000. TURP has a median hospital stay of 2 days. Significant risks may be associated: reported mortality is up to 0.25 % [21], and morbidities can include blood loss, erectile dysfunction or incontinence, resulting in considerable distress to patients. Late complications (urethral stricture and bladder neck contracture) are reported in up to 9.8 % [21]. Additional NHS costs result from delayed discharge from hospital, re-admissions and increased primary care utilisation. These unwanted consequences will increase in the future, as surgery for BPO increases in line with the ageing male population, and because most operations are conducted on older men (in 2010–11, 41 % of TURP operations were for men of 75 years or more in age). Thus, reduction in the number of surgical procedures offers direct cost savings, reduced resource use and supports the possibility of reconfigurations of surgical services. As the population ages, more operations will be considered to relieve BPO, some of which may not actually be appropriate. As a result, there is sustained interest in the diagnostic pathway.

### Expressed need

The clinical benefit of invasive urodynamics is in ensuring that surgery to relieve outlet obstruction is used only in men who actually have BPO. Thus, the NICE clinical guideline on Male LUTS [2] recommended the following research question: What is the clinical and cost effectiveness of multichannel cystometry (invasive urodynamics) in improving patient-related outcomes in men considering bladder outlet surgery? They stated that this research would clarify whether invasive urodynamics could improve the outcome of surgery, by identifying which patients have BPO. In addition, level-4 evidence indicates that men are unlikely to proceed to TURP if they are shown not to have BPO. Thus, invasive urodynamics has the potential to reduce overall numbers of surgical interventions for BPO in the NHS.

## Study aims and objectives

In men with bothersome LUTS, we hypothesise that diagnostic categorisation of BOO using invasive urodynamics improves patient selection for obstruction-relieving prostate surgery compared to a pathway with no invasive urodynamic testing. Consequently, this will make it less likely that the subgroup of men with LUTS who do not have BOO will elect to undergo surgery, thereby reducing risk of harm from surgery and potentially worse symptom outcomes.

The aim of the UPSTREAM trial is to determine whether a care pathway not including invasive urodynamics is no worse for men in terms of symptom outcome than one in which it is included, at 18 months after randomisation. This primary clinical outcome will be measured with the widely-used patient reported outcome, the IPSS at 18 months postrandomisation. We will also establish whether inclusion of invasive urodynamics reduces rates of bladder outlet surgery as a main secondary outcome.

The objectives are to answer the following questions:

- ▪ Does invasive urodynamics deliver similar or better symptomatic outcomes for LUTS measured by IPSS at 18 months after randomisation?
- ▪ Does invasive urodynamics influence surgical decision-making, as reflected in differing surgery rates in the two diagnostic pathways?
- ▪ What is the cost effectiveness of the 2 diagnostic pathways, by calculating the incremental cost per quality-adjusted life-year (QALY) gained at 18 months post randomisation?
- ▪ What are the relative harms of invasive urodynamic tests, and surgical and conservative management?
- ▪ What subsequent NHS services are required (including repeat surgery or catheterisation for acute urinary retention) for men in each arm?
- ▪ What are the differential effects on other outcomes, such as quality of life and general health?

A qualitative component has been embedded within the trial to establish patient-perceived importance of different outcomes, explore patients’ and surgeons’ perspectives on experiences of procedures and acceptable inferiority margins (the minimum difference at which surgeons would perceive there is a difference between pathways), and determine reasons for failure resulting in crossover to alternative treatment. This qualitative work will also answer the following questions:

- ▪ What is the acceptability of invasive urodynamic tests for men, and how satisfied are men with the diagnostic pathways for LUTS being tested?
- ▪ How does invasive urodynamic testing impact on decision-making for both surgeons and men with bothersome LUTS, assessed using qualitative methods?

## Methods

### Study design

**Fig. 1:**
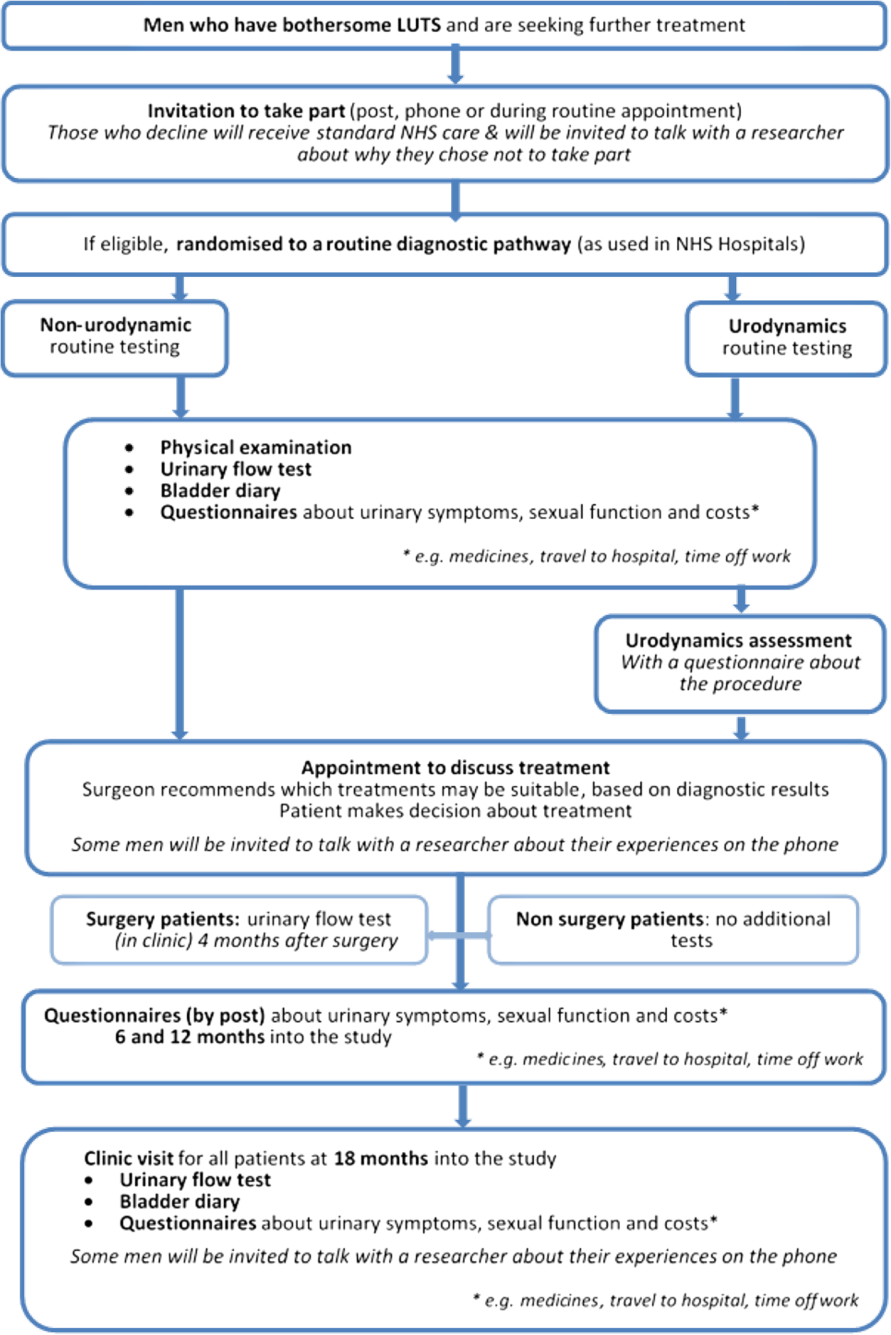
Study flow diagram

A two-arm trial randomising men with bothersome LUTS, for whom surgeons would consider offering surgery, between a care pathway based on urodynamic tests with invasive multichannel cystometry (‘urodynamics’ active intervention arm) and a care pathway based on non-invasive routine tests: i.e. without multichannel cystometry (‘non-urodynamic assessment’ control arm) (see Fig. 1, study flow diagram).

Diagnostic pathways and thresholds of testing were informed by a preliminary survey of 30 UK surgeons in 22 departments, which we undertook in 2012. This showed that the minimum baseline dataset comprises IPSS, Q_max_ with post-void bladder ultrasound scan and urinalysis.

### Setting

Urology departments of at least 26 NHS Hospitals in the UK. These currently include: Southmead Hospital, Bristol; Freeman Hospital, Newcastle upon Tyne; Royal Devon and Exeter Hospital, Exeter; Musgrove Park Hospital, Taunton; Southport and Formby District General Hospital, Southport; Kingston Hospital, Kingston upon Thames; Royal Hallamshire Hospital, Sheffield; Epsom General Hospital, Epsom; Queen Elizabeth Hospital, Birmingham; Kent and Canterbury Hospital, East Kent and Canterbury; Salisbury District General Hospital, Salisbury; Lister Hospital, Stevenage; Churchill Hospital, Oxford; The James Cook University Hospital, Middlesbrough; The Queen Elizabeth Hospital, King’s Lynn; Royal Free Hospital, London; Royal Liverpool University, and Broadgreen, Hospitals; Torbay Hospital, Torbay; Southampton General Hospital, Southampton; Kettering General Hospital, Kettering; Charing Cross Hospital, London; Royal Berkshire Hospital, Reading; Derriford Hospital, Plymouth; West Cumberland Hospital, Cumbria; Sunderland Royal Hospitals, Sunderland; and St George’s Hospital, London.

Additional sites will be identified if required.

### Participants

Men with bothersome LUTS and suspected BOO for whom surgeons would potentially offer surgery.

### Inclusion criteria

- ▪ Men seeking further treatment for their bothersome LUTS which may include surgery

### Exclusion criteria

- ▪ Unable to pass urine without a catheter (urinary retention) (excluding clean intermittent self-catheterisation (CISC) after void to empty)*
- ▪ Relevant neurological disease, such as a stroke, multiple sclerosis, Parkinson’s disease, or spina bifida (diabetes mellitus is not an exclusion criterion unless it is causing diabetic neuropathy)
- ▪ Undergoing active treatment, or on active surveillance, for prostate or bladder cancer (including low-grade/stage transitional cell cancer)
- ▪ Previous prostate surgery
- ▪ Not medically fit for surgery, or unable to complete outcome assessments
- ▪ Men who do not consent to be randomised and/or are not willing or able to comply with essential study procedures

*CISC is acceptable in situations where it is used occasionally to drain a post- void residue. However, entire reliance on CISC for all bladder emptying, or use of CISC to dilate a urethral stricture is not acceptable.

### Planned interventions

#### Baseline clinical assessment for all men

Following consent and randomisation, all men will undergo assessment as set out in the NICE clinical guideline on Male LUTS [13]:

- ▪ Assessment of general medical history to identify possible causes of LUTS, and associated comorbidities. Review of current medication
- ▪ Physical examination guided by urological symptoms and other medical conditions, an examination of the abdomen and external genitalia, and a digital rectal examination (DRE)
- ▪ Urinalysis (dipstick, or microscopy and culture)
- ▪ Urinary frequency volume chart (bladder diary)
- ▪ Measurement of urinary flow rate, with post-void residual volume measurement by ultrasound. (Note: a urinary flow rate test, recorded up to 6 months prior to date of informed consent, is acceptable to avoid unnecessary repeat for the patient)

### Discretionary tests

As this is a pragmatic trial, additional tests may be undertaken according to the usual practice of the research sites. For example, the following tests may be undertaken in line with the NICE clinical guideline on Male LUTS [13]:

- ▪ Information, advice and time to decide if they wish to have prostate specific antigen (PSA) testing if their LUTS are suggestive of BPO, or their prostate feels abnormal on DRE, or they are concerned about prostate cancer
- ▪ Cystoscopy only when clinically indicated: e.g., recurrent infection, sterile pyuria, haematuria, profound symptoms, or pain
- ▪ Imaging of the upper urinary tract when clinically indicated: e.g., chronic retention, haematuria, recurrent infection, sterile pyuria, or pain

### Interventions for randomised men

#### ‘Non-urodynamic assessment’ control arm (usual care)

Men will have clinical treatment based on the baseline clinical assessment described above.

#### Intervention arm (usual care plus urodynamics assessment)

Men will undergo the routine baseline clinical assessments set out above. In addition, they will undergo invasive urodynamics, in which catheters are used to measure bladder and abdominal pressures, during bladder filling and passing urine. Invasive urodynamics is used to calculate voiding parameters (BOO index, contractility) and assess urine storage (detrusor overactivity, bladder capacity). Hence, it should distinguish men with BOO, who should benefit from surgery to relieve obstruction, from men with reduced bladder contractility, who are unlikely to benefit from surgery, or those without obstruction with storage disorders or normal urodynamic findings.

### Method of urodynamic testing

Quality of urodynamic testing will be according to International Continence Society Good Urodynamic Practice requirements [22]. The following technical aspects of invasive urodynamic testing will be reviewed for each centre (mandatory):

- ▪ Appropriate equipment maintenance and calibration testing consistent with manufacturer instructions according to the unit log
- ▪ Measurement of bladder and abdominal pressure, including resting pressures within expected limits
- ▪ Concurrent computing of detrusor pressure
- ▪ Extrinsic filling at ‘physiological rates’
- ▪ Checks of pressure transmission (e.g. subtraction of cough impulse) during filling and after voiding
- ▪ Trace labeling for later re-interpretation; e.g. reporting of key events (e.g. detrusor overactivity, permission to void), bladder sensations and timing of ‘provocation tests’ and ‘permission to void’
- ▪ Correction for artefacts during computation of BOO and bladder contractility indices
- ▪ Correspondence of written report to symptoms and specific features of the original traces

### Surgical management

After diagnostic testing with (intervention arm) or without (control arm) urodynamics, patients will see their surgeon to decide on whether to proceed to surgical treatment. The treatment decision is between the urologist and the patient and there are no treatment ‘requirements’ imposed by the UPSTREAM study. We aim to capture urologist and patient opinions about treatment decisions in the relevant case report form(s) (CRF). As a pragmatic trial, standard practice for the centres will be followed, relating to type of surgery (providing it is a NICE-approved surgical procedure, e.g. monopolar or bipolar TURP, or laser), whether to stay on LUTS medications, antibiotic prophylaxis and other factors. Type of surgery will be recorded. All conservative and surgical management plans and actual treatment received will be documented. As this is a pragmatic study, surgeons may feel it necessary in some cases to conduct additional tests outside of the participant’s allocated intervention group. Centres are asked to record, in the baseline CRF, whether the participant received the diagnostic assessments that they were randomly allocated to, and provide reason(s) if assessment was different to that allocated. As we are recording assessments received versus assessment allocation in trial document, such a deviation would not require additional ‘Protocol Noncompliance’ reporting.

All other Good Clinical Practice (GCP) and/or protocol deviations should be recorded on the ‘GCP/Protocol Noncompliance Report Form’ (provided in the Site File) and forwarded to the Trial Manager who will notify the Chief Investigator (CI) and Trial Sponsor.

### Allocation to trial groups

All eligible and willing men will be randomly allocated to receive one of two assessment pathways, as outlined above; that is either a) usual care (non-urodynamics, control); or b) usual care plus urodynamics assessment (intervention).

All men who enter the study will be logged with the central trial office and given a unique six-digit study (participant) identification number. Randomisation will utilise the existing proven remote automated computer randomisation application at the study administrative centre in the Bristol Randomised Trial Centre (BRTC, a fully registered UK CRN clinical trials unit) in the University of Bristol. The randomisation application will be available to participating centres, both as a telephone-based interactive voice response (IVR) system and as an Internet-based service, for them to complete the randomisation procedure themselves, on site.

Further details of ‘Identification, Recruitment and Consent’ are outlined below (page 10).

### Study outcome measures

The measures have been selected according to the specifications of the HTA commissioning brief.

#### Primary outcome measure

- ▪ Primary clinical outcome: difference in lower urinary tract symptom (LUTS) between the 2 arms at 18 months (post randomisation), measured with the IPSS. IPSS is validated [23], well-known and widely used in the NHS.

#### Secondary outcome measures

- ▪ Surgery rate (the relative proportion of men in each group having surgery up to 18 months after randomisation)
- ▪ Cost-effectiveness analyses from the perspectives of the NHS, personal social services and patients. Subsequent need for surgery (related to their LUTS) during any stage of the trial will be recorded
- ▪ Adverse events of testing and treatment (e.g. infection, urinary retention)
- ▪ Measures from the International Consultation on Incontinence Questionnaires (ICIQ) [24] will be used alongside the IPSS, giving sensitive and comprehensive assessment of LUTS severity/bother, sexual function, quality of life and satisfaction with urodynamic testing. The following will be measured at 6, 12 and 18 months:
- ▪ IPSS
- ▪ ICIQ Male LUTS (ICIQ-MLUTS)
- ▪ ICIQ sexual function in Male LUTS (ICIQ-MLUTS-sex)
- ▪ ICIQ urodynamics satisfaction (ICIQ-UDS-S) will be administered at a single time point after urodynamic testing for the interventional arm
- ▪ Q_max_ at 18 months. In men undergoing surgery in both arms, an additional Q_max_ measure at 4 months after operation will be used as a quality measure for surgery
- ▪ The EuroQol Group’s 5 dimension health status questionnaire (EQ-5D-5 L) will be used to provide the quality of life weights used to calculate QALYs
- ▪ Qualitative interviewing will explore user acceptability and influences on decisions made by the participating men and the surgeons

In addition, all men will be invited to consent to long-term follow-up, including use of computerised NHS records, Hospital Episodes Statistics (HES) data and other routine data sources.

### Participant entry

#### Identification, recruitment and informed consent

All eligible men referred with voiding LUTS will be identified by the consultant, dedicated research nurse, or designated team member at time of receipt of referral letter or during patients’ clinical appointments. Hospital staff should complete trial-specific screening logs for all potentially eligible men and provide confirmation of the patient’s outcome for the study; this will be one of three: 1) patient confirmed as ineligible; 2) patient was eligible but declined to take part; and 3) patient was eligible and consented to take part. These will be reviewed by the UPSTREAM Office Team (BRTC) on a monthly basis.

Due to variation in patient pathways in each hospital, these arrangements should be individualised according to local circumstances in each site. Those patients identified from referral letters will be sent a Patient Information Sheet (PIS), Assessment Information Sheet (AIS) and covering letter. Alternatively, the research nurse will describe the study to the patients at their clinical appointment and, if interest is expressed, provide further details of the study by means of the PIS and AIS. An approved study-specific poster can also be displayed in suitable clinic rooms, which provides the contact details of trial-related staff that interested men can contact for further information.

*If the patient agrees to the study*, they will be given a chance to ask questions and should ideally have at least 24 hours to think about taking part before being consented and randomised. Written informed consent will be obtained from all patients who agree to take part in the study. The PIS and the consent form will refer to the possibility of long-term follow-up and being contacted about other research if the man is willing. If the patient is happy to take part in the study without having been given PIS and study details over the previous 24 hours, and requests to provide written consent and complete baseline questionnaires at that time (i.e. to avoid having to return to the hospital for an additional appointment) this is possible. In such cases we suggest that the research centre completes written consent and baseline questionnaires, there and then, but does not randomise the patient until at least 24 hours have passed. After at least 24 hours, the centre should contact the patient by telephone to confirm whether they are still willing to proceed with the study, and if so, the centre can proceed with randomisation and inform the patient of his intervention allocation via the telephone. If the patient has changed his mind, however, and no longer wishes to be randomised, the research centre should complete the ‘UPSTREAM Change of Permissions/Withdrawal Form’ accordingly, and follow essential reporting procedures specified on the form. For clarity, a copy of the consent form and completed Change of Permissions/Withdrawal Form should be kept at site, as well as forwarded to the UPSTREAM Office Team for records. All other data collected for such a patient, however, such as baseline questionnaires, should be suitably discarded by the research site; the trial has no need to retain this information as the patient has decided not to enrol (be randomised) into the trial. For men who are randomised, the research centre should also record that the patient opted for this consent and randomisation approach in their medical notes, and in the UPSTREAM Baseline CRF (Comments section). This alternative consent and randomisation process helps the patient to avoid returning the hospital simply for the purpose of the trial.

*Men who are not willing to be randomised, but who would otherwise be eligible*, will be asked to consent to being contacted for qualitative research to explore reasons for non-participation.

*All men who enter the study* will be logged with the central trial office and given a unique six-digit study (participant) identification number, and randomised. Hospital staff will complete and send a study approved letter to the participant’s General Practitioner (GP) informing them that their patient has entered into the trial.

Hospital staff will be informed about the study by the Principal Investigator (PI) and the research nurse, so that they can answer queries from participants and their relatives.

### Withdrawal criteria

Participants will remain in the trial unless they choose to withdraw or if they are unable to continue for a clinical reason. If a participant withdraws consent, further participant questionnaires will not be collected. However, permission will be sought for the research team to continue to collect outcome data from their health care records. Participants are informed in the PIS that they have the right to withdraw all personal data held by the study. Study specific procedures for a participant’s change of permissions, or withdrawal, are outlined in the relevant trial working guidelines.

### Randomisation, blinding and prevention of bias

#### Randomisation

All men who enter the trial will be logged with the central study office and given a unique, six-digit study (participant) identification number. Randomisation will utilise the existing proven remote automated computer randomisation application at the study administrative centre in the BRTC. Participants will be randomly allocated to treatment arms using an automated web/telephone randomisation system provided by the BRTC. Randomisation will be stratified by centre.

#### Blinding

Blinding in the urodynamic unit is not possible nor appropriate in this pragmatic trial, given that men are only catheterised in the invasive group, hence group allocation cannot be concealed from the man or the staff. We do not feel it is necessary or ethical to perform sham catheterisation to conceal the nature of testing. Furthermore, knowledge of the results of urodynamic testing underpins the urologist’s ability to make a management decision, in conjunction with his patient, so neither the man nor his urologist can be blinded to the intervention or its findings. However, outcome assessment is largely by participant self-completed questionnaire, so avoiding interviewer bias.

### Methods to protect against bias

#### Urodynamic techniques

We will centrally monitor deviations from agreed protocols and review > 10 % traces from each research centre. All investigators are already experienced urodynamics investigators, or work with an experienced urodynamics unit meeting the national minimum standards.

#### Standardisation of surgical techniques

All investigators are already experienced prostate (TURP) surgeons. The research nurses and the surgeons will complete a Peri-Operative CRF at the time of surgery, including any intra-operative difficulties or complications. As this is a pragmatic trial, surgical procedure and post-operative care will be according to local centre practice.

### Loss to follow-up (attrition bias)

Loss to follow-up in our previous trial of conservative treatment for men with urinary incontinence after prostate surgery [25] was 5 to 10 % at 1 year. However, a more conservative estimate of just over 20 % loss to follow-up has been used in the sample size calculations. We will take very active measures to minimise loss of men from the study in line with Research Ethics Committee approval, such as reminder letters, phoning/texting/ emailing the men, obtaining back-up ‘best contact’ addresses, using non-contingent retention incentives [26], and checks with their GPs [27]. In addition, we will obtain consent from the men to enable us to access centrally-held NHS data; e.g., via the NHS Strategic Tracing Service in England and Wales to find new addresses, and electronic data linkage which records any in-patient episodes and outpatient visits.

### Other sources of bias (detection bias)

Where feasible, research staff will be blinded to allocation while conducting data collection for outcomes, performing data entry and analysis, and by using study numbers only to identify men, questionnaires and diaries. Men will be asked not to reveal information about their diagnostic evaluation and treatment. Staff will be asked in the 18-month CRF to record whether or not they knew which group the man had been allocated to, and hence which diagnostic tests were performed before undertaking outcome assessments. All men will be actively followed up, with analysis based on the intention-to-treat principle. All analyses will be clearly pre-defined to avoid bias.

### Adverse events

#### Adverse event (AE)

An AE includes any untoward medical occurrence in a study participant, including abnormal laboratory results, symptoms or a disease that does not necessarily have a causal relationship with procedures required by the protocol. In all instances it will be up to the physicians responsible for the participants’ care to determine whether the person’s change in health is *related* to the trial.

AEs are not:

- ▪ continuous and persistent disease or symptom, present before the trial, which fails to progress
- ▪ signs or symptoms of the disease being studied; or
- ▪ treatment failure

For the UPSTREAM study, pre-planned hospitalisation or elective procedures: e.g. for pre-existing conditions which have not worsened, does not constitute an AE. However, any hospitalisation of a pre-existing condition resulting from worsening, or elective procedures booked after the patient has signed the consent form would constitute an AE.

### Serious adverse event (SAE)

An adverse event is defined as ‘serious’ (SAE) if it:

- ▪ results in death of the participant
- ▪ is life-threatening: the term ‘life-threatening’ refers to an event in which the participant was at risk of death at the time of the event; it does not refer to an event which hypothetically might have caused death if it were more severe
- ▪ requires hospitalisation, or prolongation of existing inpatient hospitalisation
- ▪ results in persistent/significant disability/incapacity
- ▪ is otherwise considered medically significant by the investigator*

*Important AEs that are not immediately life-threatening or do not result in death or hospitalisation but may jeopardise the subject or may require intervention to prevent one of the other outcomes listed in the definitions above, may also be considered serious. Medical judgment will be exercised in deciding whether an AE is serious in other situations.

### Expected, related adverse events

Within UPSTREAM, an AE is defined as ‘related’ if it occurs as a result of a procedure required by the protocol, whether or not this procedure is the specific intervention under investigation and whether or not it would have been administered outside the study as normal care.

The following events are expected, related AEs during/after any diagnostic procedures:

- ▪ urinary tract infection
- ▪ bacteriuria
- ▪ haematuria
- ▪ urinary retention
- ▪ discomfort
- ▪ dysuria
- ▪ urethral trauma

The list below itemises the expected, related AEs summarised from the literature for prostate surgery:

- ▪ excess blood loss (> 500 ml)
- ▪ blood transfusion
- ▪ urethral injury
- ▪ bladder injury
- ▪ bowel injury
- ▪ injury to blood vessels or nerves
- ▪ anaesthetic complications
- ▪ thrombosis/deep vein thrombosis/pulmonary embolism
- ▪ prolongation of post-operative catheterisation
- ▪ recatheterisation
- ▪ urinary tract infection
- ▪ other infection (sepsis, septicaemia, abscess)
- ▪ new urinary tract symptoms
- ▪ constipation
- ▪ discomfort/pain
- ▪ new sexual problems
- ▪ death

Complication rates will be recorded and classified using the internationally accepted Clavien-Dindo classification in trial CRFs [28].

### Reporting procedures for adverse events

Within UPSTREAM, all adverse (serious and non-serious) events should be recorded on the ‘UPSTREAM Adverse/Serious Adverse Events’ form, whether originally notified on a CRF, participant questionnaires or by other means. In addition, all deaths with any cause (related to the trial or otherwise) should also be recorded on the ‘UPSTREAM Adverse/Serious Events’ form.

All AEs should be reported to the UPSTREAM Study Office Team; depending on the nature of the event the reporting procedures below should be followed.

### Non-serious adverse events

All AEs, whether expected or not, should be recorded using the ‘UPSTREAM Adverse/Serious Adverse Events’ form. The UPSTREAM online database should also be updated accordingly by the local research centre at the earliest opportunity. A copy of the completed form should be kept in the Site File and in the patient’s notes.

### Serious adverse events (SAEs)

Local PI or Research Nurse: *all SAEs* including deaths from any cause (related or otherwise) should be recorded on the ‘UPSTREAM Adverse/Serious Adverse Events’ form, whether originally notified on a CRF, participant questionnaires or by other means. The completed form should then be forwarded to the Trial Manager within 24 hours of learning of a SAE, or within 24 hours in the event of death. Detailed reporting procedures for SAEs are found in trial specific working guidelines.

Chief Investigator (CI)/or Trial Manager:

- ▪ The Trial Manager will inform the CI of all SAEs. If, in the opinion of the local PI and the CI, the event is confirmed as being *serious* and *related* and *unexpected*, the CI or Trial Manager will notify the sponsor within 15 days (24 hours in the event of death) of receiving the AE notification. The sponsor will provide an assessment of the SAE
- ▪ The CI (or Trial Manager) will report any *related* and *unexpected* SAEs to the main Research Ethics Committee and the Data Monitoring Committee (DMC) within 15 days of the CI becoming aware of it
- ▪ All related SAEs will be summarised and reported to the Ethics Committee, the Funder, the DMC and the Trial Steering Committee (TSC) in their regular progress reports

### Assessment and follow-up

#### Clinical outcomes

Clinical outcomes will be assessed by participant-completed questionnaires at baseline, 6 months (postal (or online or by telephone if required)), 12 months (postal (or online or by telephone if required)) and 18 months post randomisation (clinic appointment). Free flow rate testing (maximum flow rate, voided volume (VV), post-void residual) will be used at baseline and 18 months; an additional measurement of flow rate in men undergoing surgery in both groups at 4 months after surgery (± 1 month) will provide objective assessment of effective relief of BOO. The components and timing of follow-up measures are shown in Table 1.

**Table 1:** Measurement outcomes table: components/timing

	Baseline	Urodynamics	Peri-operative	4 mths post surgery	6 mths	12 mths	18 mths
	All pts	UDS pts	Surgery pts	Surgery pts	All	All	All
CRFs	●	●	●	●			●
IPSS	●				○	○	●
ICIQ-MLUTS	●				○	○	●
ICIQ-MLUTSsex	●				○	○	●
ICIQ-UDS-S		●					
Flow rate/PVR	●			●			●
EQ-5D-5 L	●				○	○	●
Resource use questionnaire	●				○	○	●
Bladder diary	●						●
Case note review							⋄
Qualitative interview* (selected patients)		1 week after treatment decision					At end of treatment
Qualitative interview (staff)							●

● Clinic/Hospital; ○ Postal; ⋄ Hospital sources; *at home or by telephone

*CRF* case report form, *EQ-5D-5 L* EuroQol Group’s 5 dimension health status questionnaire, *ICIQ-MLUTS* International Consultation on Incontinence Questionnaires Male lower urinary tract infections, *ICIQ-MLUTSsex* International Consultation on Incontinence Questionnaires sexual function in Male LUTS, *ICIQ-UDS-S* International Consultation on Incontinence Questionnaires urodynamic satisfaction; *IPSS* International Prostate Symptom Score, *mths* months, *pts* patients, *PVR* post-void residue, *UDS* urodynamic studies

Where possible, telephone interviews will be conducted with men who withdrew or declined randomisation.

Steps will be taken to minimise loss to follow-up, including reminder letters and telephone calls. In particular, the primary outcome measure (IPSS at 18-months post randomisation) could be collected via the telephone if necessary.

### Economic data collection

Resources used in relation to the management of bothersome LUTS will be measured from randomisation to 18-month follow-up. The CRFs will be used to measure: the initial hospital resource use during the diagnostic phase of the trial; the perioperative stay for those men who subsequently undergo surgery and, with the exception of this surgery, any in-patient stays, out-patient visits and procedures occurring at the treating hospitals, where the study has research governance approval, from the end of the diagnostic phase until the end of the 18 month follow-up period for any man who has had any non-routine follow-up hospital care, as identified at the 18-month clinic follow-up. The CRFs will be designed so that the resource use collected can be costed using NHS tariffs. At baseline, 6 and 12 months follow-up the men will be given a study designed Resource Use Log (RUL) to be used as an aide memoire in which to record prospectively NHS hospital and community-based health care use, medications, social service resource use, time off work and any other expenses resulting from their treatment. The baseline RUL (0–6 months) will be given to the patients at the baseline assessment clinic; all subsequent RULs will be posted by the UPSTREAM Office Team. These logs will reflect the design of the 6, 12 and 18-month resource use questionnaires respectively. At the baseline and 18-month follow-up clinic appointments, resource use questionnaires will be interviewer-administered if time permits, otherwise the questionnaires will be given to the men at the clinics for them to complete in their own time, and return them by post if necessary. At 6-month and 12-month follow-up, self-completed resource use questionnaires will be posted to the men for them to complete, using the information from the RUL.

The EQ-5D-5 L will be included within the questionnaires given to all men at baseline, 6, 12 and 18 months follow-up.

### Medical record abstraction

At 18 months follow-up, in-patient stays, out-patient visits and procedures relating to the man’s urinary symptoms, identified though the 18-month resource use questionnaire occurring in the treating hospitals, where the study has research governance approval, will be abstracted from the patients’ medical records. In such a way that the resource use collected can be costed using NHS tariffs.

### Qualitative data collection

The aim of the study is to understand patients’ and health care professionals’ views and experiences of invasive urodynamic testing for male BOO and BOO surgery.

Objectives:

- ▪ To explore through qualitative methods patients’ views, experiences and beliefs about LUTS
- ▪ To examine patients’ understanding and knowledge of testing for BOO and treatment expectations
- ▪ To understand patients’ and health care professionals’ experiences of the trial, including their experience, opinions, acceptability and feasibility of invasive urodynamic testing/non-urodynamic assessment
- ▪ To investigate patients’ and health care professionals’ decision-making regarding surgery for male BOO
- ▪ To use qualitative methods to understand barriers and facilitators to invasive urodynamic testing
- ▪ To explore the information and support needs of patients and health care professionals in relation to invasive urodynamic testing and BOO surgery
- ▪ To investigate patients’ and health care professionals’ experiences, attitudes and opinions regarding male BOO surgery and recovery

### Overview

In order to examine the views and experiences of invasive urodynamic testing for male BOO we will conduct in-depth semi-structured qualitative interviews with patients and health care professionals involved in their care. Qualitative findings will help to illuminate the perceived effectiveness and acceptability of invasive urodynamic testing for male BOO, its impact on clinical decision-making and explore any barriers to their uptake outside of the trial.

Qualitative methods have been chosen as the most appropriate means to achieving a deep understanding of beliefs and perceptions of key medical events [29, 30]. Interviews allow for the exploration of complex and sensitive issues, allowing participants to engage in a dialogue in their own language and drawing on their life experiences to explore the issues which are important to them.

Previous studies have successfully utilised qualitative methods to investigate patients’ views, experiences and health beliefs about LUTS [31–34], triggers and barriers to help seeking [35] perspectives on treatment outcomes [36]. However, to our knowledge to date no studies have examined patients’ and health care professionals’ views and experiences regarding invasive urodynamic testing for BOO. We are combining qualitative methods and controlled trial methods as has long been advocated [37].

### Study design

In-depth interviews [38] will be conducted with trial participants (from all arms of the trial). Purposive sampling will ensure that adequate numbers of interviews will be conducted with men from each of the possible randomised groups according to treatment allocation. Firstly, participants will be interviewed 1 week after a decision has been made regarding treatment. These interviews will consider and compare their views and experiences of the trial, explore participants’ experiences of LUTS, understanding and knowledge of BOO, views and experiences of invasive urodynamic testing, decision-making regarding surgery and information and support needs.

A second interview will be conducted with a second group of participants 18 months after randomisation (after treatment has been completed) to additionally explore views and experiences of treatment and recovery.

Telephone interviews will be conducted with a sample of men who withdrew from the trial. In addition, telephone interviews will be conducted with those who declined to be randomised in the trial. Health care professionals will also be interviewed at the end of the trial to gather data on their views and experiences of assessment with and without urodynamics, information and support needs and their attitudes to its future implementation.

Health care professionals (e.g. urologists, urodynamics technicians, nurses, etc.) involved in the trial will be purposively sampled in relation to (i) the trial site and (ii) length of time since qualification.

The sample sizes will be determined by the need to achieve data saturation, such that no new themes are emerging from the data by the end of data collection [39]. Interviews will be analysed in batches, and sampling will continue until no new themes are emerging from the interviews. This is likely to include up to 30 health care professional and 45 face-to-face trial patient interviews and 20 telephone interviews with those that declined trial participation or withdrew from the trial.

The sampling frame is shown in Table 2.

**Table 2:** Sampling frame

Arm	Urodynamic testing				Non-invasive testing				Total
Treatment	Surgery		Conservative		Surgery		Conservative		
IPSS/ICIQ-MLUTS storage subscales	High	Low	High	Low	High	Low	High	Low	
Pre treatment (after treatment decision)	4	4	4	4	3	3	3	3	28
Post treatment	4	4			4	4			16
Total trial interviews (to include a mix of older/younger, randomised groups receiving surgery or conservative treatment and location (weighted towards Bristol)									44
Telephone interviews with decliners and withdrawals (approximately 20 minutes each)									20
Grand total									64

*ICIQ-MLUTS* International Consultation on Incontinence Questionnaires Male lower urinary tract infections, *IPSS* International Prostate Symptom Score

### Interview conduct

A flexible topic guide will be used in order to assist questioning during in-depth individual interviews. The topic guide will be devised to ensure that the primary issues are covered across all interviews, but do not dictate data collection. The topic guide will incorporate considerable flexibility to enable participants to introduce new issues unanticipated by the researchers. Topic guides will be modified as necessary throughout the course of the study to reflect findings as they emerge. The researcher will use open-ended questioning techniques to elicit participants’ own experiences and views of key events and participants will be asked to provide examples. Interviews will be recorded, transcribed and anonymised to protect confidentiality.

### Data analysis

Interview transcripts will be checked for accuracy and then imported into NVivo qualitative data analysis software (QSR International, Daresbury, UK) which aids the management and indexing of qualitative data. Analysis will inform further data collection: for instance, analytic insights from data gathered in earlier interviews will help identify any changes that need to be made to the topic guide during later interviews.

Thematic analysis [40], utilising a data-driven inductive approach [41], will be used to scrutinise the data in order to identify and analyse patterns and themes of particular salience for participants and across the dataset using constant comparison techniques [42, 43].

Firstly, the transcripts will be read several times, to gain familiarisation with the data and initial ideas noted. The transcripts will then be examined on a line-by-line basis with inductive codes being assigned to the segments of the data that provide insight into the participants’ views and understanding of their experiences. An initial coding frame will be developed and new data will be compared initially to previous data, and then to the properties of emerging categories that contain the main themes. The process of constant comparison will allow for the generation of new themes, re-classify themes and incorporating themes within other themes [42, 43] and the coding frame will be modified, if needed, as analysis develops. The data will be scrutinised for negative cases and reasons for the deviance will be explored by comparison with the whole dataset.

Transcripts from the patients’ and health care professionals’ interviews will be analysed separately, with coding frames being developed for each separate phase of the research. A subset of transcripts will be independently double-coded by other members of the research team and compared; any discrepancies will be discussed within the research team and resolved in order to achieve a coding consensus and to ensure robust analysis.

### Data management and security – overall trial

#### Data collection and transportation

All data held in Bristol will conform to the University of Bristol Data Security Policy and in Compliance with the Data Protection Act 1998. Data collected on the paper CRFs at study centres or as questionnaires from participants will be identifiable only by participant study number. Information capable of identifying individuals and the nature of treatment received will be held in the database with passwords restricted to UPSTREAM study staff.

Audio recordings of qualitative data made during the interviews will only refer to the participant by their study number.

### Retention of data

Patient identification codes will be held by BRTC for 15 yearsand all other data sources will be stored for 10 years after the close of the study. Personal data (e.g. name and address, or any data from which a participant might be identified) will be withdrawn from the study if this is requested by a participant.

### Access to the data

The Senior IT Manager (in collaboration with the CI) will manage access rights to the data set. Prospective new users must demonstrate compliance with legal, data protection and ethical guidelines before any data are released. We anticipate that anonymised trial data will be shared with other researchers to enable international prospective meta-analyses.

### IT security

All IT systems supported and maintained by the University of Bristol Information Services will have infrastructure including server and server-based applications and desktop system maintenance. All NHS IT systems will be similarly supported. Data is stored centrally on robust data systems with file versioning and recovery and mirroring on a second site. The BRTC Randomisation system infrastructure is also maintained by University Information Services.

### Auditing and inspection

The study may be subject to inspection and audit by North Bristol NHS Trust (NBT) under their remit as sponsor, and other regulatory bodies, to ensure adherence to GCP and the NHS Research Governance Framework for Health and Social Care (2nd edition).

### Statistics and data analysis

#### Sample size determination

We decided that the important consideration is that the group of men randomised to having urodynamics should have clinical outcomes which are not inferior (rather than equivalent) to those who are randomised to management without urodynamics. This is because the likely reduction in surgery rates in the former group due to more accurate diagnosis should not disadvantage them in terms of clinical improvement. We therefore calculated our sample size based on both the primary outcome and surgery rates: non-inferiority of symptoms at 18 months after randomisation; and a reduction in surgery rates in the intervention arm.

In Bristol, audit data for 5670 men presenting with LUTS suggestive of poor or obstructed urine flow show that 73 % to 83 % would have surgery. If an invasive urodynamics test was conducted on the same men, the data indicate that surgery would only be carried out in 60 %, based on the prevalence of impaired bladder contractility contraindicating surgery. Using the more conservative difference we expect the intervention to reduce surgery from 73 % in non-urodynamic assessment to 60 % in the intervention arm.

Symptom scores will potentially improve for those men in both arms who undergo appropriate surgery. Symptomatic outcome of surgery is confounded by a number of factors for which we cannot control. These include:

- ▪ Long-standing BPO might impair bladder contractility, reducing the symptom benefit of surgery
- ▪ BPO-relieving surgery increases the calibre of the outlet channel regardless of whether BPO is present, and this might improve urinary stream in men who technically did not have BPO
- ▪ A ‘placebo effect’ is known to arise both from clinical contact and from the surgical procedure
- ▪ Whilst voiding LUTS (obstruction) typically improves after surgery, this advantage will be offset in some men due to deterioration in storage LUTS (e.g. incontinence, overactive bladder)

We therefore anticipate the overall IPSS at 18 months in both arms might be similar despite group differences in surgery rates.

However, to ensure that the men in the urodynamic arm are not disadvantaged by the reduction in surgery rates, we need to ensure that the primary outcome, symptom score, has adequate power to rule out non-inferiority. Therefore, assuming no difference between the groups, a trial of 310 men per arm will give 80 % power to rule out a non-inferiority margin of 1 point below the mean IPSS in the non-urodynamic arm, using a 1-sided *t* test (common standard deviation [SD] of 5) at the 5 % significance level. We have chosen 1 point on the IPSS as it is a conservative estimate of the level at which we would assume non-inferiority, since a change of 2 points is associated with a change in global impression in urinary condition [44].

Loss to follow-up in our previous trial of conservative treatment for men with urinary incontinence after prostate surgery [25] was 5 to 10 % at 1 year. However, a more conservative estimate of just over 20 % loss to follow-up has been used in the sample size calculations. Therefore, sample size will be 388 per arm to take into account 20 % loss to follow-up. Our recruitment target will be 400 per arm with the aim of achieving no less than 388 per arm at 18 months follow-up.

### Statistical analysis

Baseline characteristics will be examined at baseline to ensure randomisation has provided the two pathways with patients that are comparable on equal terms. Any differences in excess of 0.5 SDs or 10 % or more will be controlled for in sensitivity analyses to ensure that the imbalance does not affect the overall result. If it does then both the adjusted and unadjusted results will be quoted in future reports and papers.

The analysis and reporting of the UPSTREAM trial will be in accordance with Consolidated Standards of Reporting Trials (CONSORT) [45] guidelines. All analyses will be on an intention-to-treat (ITT) basis where men are assessed in the groups to which they were assigned at randomisation. For all analyses carried out, effects estimates will be presented along with confidence intervals and *p* values. All outcomes will be described and compared with the appropriate descriptive statistics where relevant: mean and SD for continuous and count outcomes, medians and inter-quartile range if required for skewed data and numbers and percentages for dichotomous and categorical outcomes (e.g, subjective recurrence of incontinence).

### Analysis of the primary outcome

The primary outcome will be the IPSS score at 18 months post randomisation. This outcome is on a continuous scale and consists of 7 questions concerning urinary symptoms with a 6- point Likert scale response from 0–5; therefore, with a minimum score of 0 and maximum score of 35. The difference between the scores for the two pathways will be evaluated using linear regression, adjusting for centre and IPSS score at baseline. To assess non-inferiority of IPSS the post-treatment difference at 18 months between the 2 arms will be used with a non-inferiority margin of 1.0. Between-centre effects will be examined and a mixed model approach with treatment group as a fixed factor and investigational site as a random effect will be considered.

The primary analysis will be based on the observed data supported by a sensitivity analysis where all missing data will be imputed at baseline using appropriate imputation methods and a range of assumptions. A sensitivity analysis will also be conducted to assess the treatment effect for those who fully comply with the intervention.

### Analysis of secondary outcomes

All secondary outcomes listed in the ‘outcomes’ section will be analysed using appropriate regression models, adjusting for centre and the baseline measure of the outcome (where possible). The main secondary outcome for this trial is the uptake of surgery in each pathway; this will be analysed using logistic regression. Other symptom scores such as the ICIQ-MLUTS, which is potentially more sensitive but less widely recognised, will be evaluated in a similar way to the IPSS primary outcome; adjusting for centre and baseline scores. Missing items on the health-related outcome measures will be treated as per the instructions for that particular measure and imputed if necessary. Acute urinary retention as a possible complication will also be examined as secondary outcome.

### Planned further analyses

The effects of urodynamics may be more pronounced in groups of patients with certain characteristics. Subgroup analyses will, therefore, be carried out to assess the difference in treatment effect for pre-specified factors. These subgroup analyses will be carried out on the primary analysis (IPSS score) and main secondary outcome (surgery rate). Formal tests of interaction between the dichotomised variables and treatment pathway will be carried out to test whether treatment effect differs between the different subgroups of patients.

Of note, subgroup analysis will be carried out for men presenting with more and less substantial storage LUTS (urgency, increased frequency and nocturia), based on the IPSS storage subscore and/or the ICIQ MLUTS storage score.

Subgroup analysis will be undertaken for the differing clinical diagnoses reached at the ‘Clinical Decision’ stage; all of the factors below are on a Yes/No basis:

- ▪ Voiding dysfunction due to BOO, with or without reduced bladder contractility
- ▪ Voiding dysfunction due to reduced bladder contractility, with or without BOO
- ▪ Storage dysfunction (overactive bladder syndrome/detrusor overactivity)
- ▪ Storage dysfunction (nocturia)

### Proposed frequency of analyses

Men will be followed up at 6 months (by post), 12 months (post), and 18 months (clinic), after randomisation. Men undergoing surgery will also attend clinic for flow rate testing 4 months after operation. They will be asked to consent to longer term follow-up although this is not funded. The main analysis will be performed when all 18-month follow-ups have been completed. An independent Data Monitoring Committee will review confidential interim analyses of accumulating data at its discretion.

### Economic evaluation

The trial will include a formal economic evaluation comparing the costs and cost-effectiveness of the interventions from the perspectives of the NHS, personal social services and patients. The cost of the interventions and the use of primary and secondary NHS services by the men, personal and social service costs, costs to the men arising from their treatment (e.g. over-the-counter medication) will be estimated through the collection of resource use data as outlined earlier and the valuation of these data.

NHS tariffs will be used to quantify the resource use information contained in the CRFs. All other resource use will be valued using routine sources and information from the patients themselves.

Differences in costs between the arms from each of the three perspectives will be evaluated using regression techniques adjusting for pre-specified baseline characteristics, randomisation variables and a centre effect. The same model specification will be used to evaluate the differences in QALYs.

For each of the three perspectives the difference in costs and in effectiveness in terms of surgery rates and IPSS scores will be examined. If neither arm is dominant: i.e. both cheaper and more effective, then incremental cost-effectiveness ratios will be calculated in relation to surgery rates and IPSS scores. The differences in costs and QALYs will be examined using the net benefit framework over a range of values for the QALY. This will facilitate the use of regression modelling to adjust for pre-specified baseline characteristics, randomisation variables and centre effects.

Uncertainty for all these analyses will be addressed using cost-effectiveness acceptability curves and sensitivity analyses. One aspect of uncertainty is likely to be that of missing data. In order to address this, a pre-specified analysis plan will be created in which the plausible assumptions about missing data will be created. These assumptions will then be tested within the sensitivity analyses.

### Internal feasibility phase and recruitment rates

An internal feasibility phase, intended to verify that recruitment is possible, was first conducted in 4 centres between months 7 and 9 (October–December 2014). Delays in centres being ready for the trial and additional work to improve recruitment amongst centres that were ready meant this initial verification window was too narrow. It was estimated that the equivalent of 12 recruitment months (4 centres × 3 recruiting months each) should yield 48 participants out of the overall accrual target of 800; however, a more realistic assessment of actual recruitment time meant a revised target of 17 patients, of which we recruited 13. The initial cumulative accrual prediction was based on a relatively simple linear trend assumption without incorporating differential recruitment over time by centres or different recruitment rates within centres. Therefore, at the request of the TSC, we revised the accrual projections based on a more realistic assumption conditioned both on differential recruitment by centres over time and recruitment capacity.

Subsequently, a further internal feasibility phase was conducted between February and April 2015 with a revised target of 42 participants from 4 centres, with additional reporting to the TSC upon conclusion to gain a realistic assessment of recruitment. Between 1 February and 30 April the 4 centres recruited 58 participants, exceeding the agreed target by 16.

Strategic findings from the second feasibility phase, and implications for the trial overall, are outlined in the discussion section of this paper (pages 23–24).

### Patient panel – contribution of patient and public involvement (PPI)

An expert panel (patient panel (PP)) of service users will be invited to advise as we proceed through the preparation for, and the main recruitment period of, the study. The PP will be invited to meet with the CI and/Project Management Group (PMG) on a monthly basis in the setting-up phase and at the conclusion of the study, and quarterly at other times (or more often if needed). Their guidance during the preparation of the patient consent documentation, including information, will be vital for the success of the consent and randomisation processes. They will also help ensure appropriate approaches to the delivery of the diagnostic/treatment pathway, especially the approach to the doctor/patient decision step. They will review communications in respect of clarity and avoidance of potential ambiguity. We will also seek advice from the PP on the reporting and dissemination of findings amongst relevant patient groups, such as the Bladder and Bowel Foundation.

### Study coordination in Bristol (BRTC)

The Study Office will be based in the BRTC within the School of Social and Community Medicine at the University of Bristol, and will provide day-to-day support for the clinical centres. The Trial Manager based at the BRTC will take responsibility for the day-to-day supervision of study activities. The Study Administrator will provide clerical support to the trial, including organising all aspects of the postal questionnaires (mailing, tracking, and entering returned data). As per BRTC’s business and costing model, the Senior IT Manager will oversee all IT aspects of the study, while the Senior Trials Manager will provide mentoring and guidance to the Trial Manager and advice to the team on generic coordination issues. The BRTC Quality Assurance Manager will oversee and demonstrate that BRTC’s standard operating procedures for trials have been followed and properly documented, including observance of GCP throughout.

The UPSTREAM Study Office Team will meet formally at least monthly during the course of the study to ensure smooth running and trouble-shooting.

### Project Management Group (PMG)

The study will be supervised by a PMG. The chair of this group will be the CI and will consist of grant holders, representatives from the Study Office and a representative from the PP. The PMG will meet monthly for the first 6 months from study start and quarterly thereafter. In addition, the PMG will also meet at the Trial Steering Committee meetings.

### Trial Steering Committee (TSC)

The role of the TSC is to monitor and supervise the progress of the trial. The TSC will consist of a chair and at least two other independent members, and also the Trial Manager and the CI. The PP of service users and the HTA will be invited to nominate a representative to sit on the TSC. Other non-voting members will include the grant holders. Observers may also attend, as may other members of the PMG or members of other professional bodies at the invitation of the Chair.

### Data Monitoring Committee (DMC)

The DMC will also have an independent chair and at least two other independent members, and will monitor accumulating trial data during the course of the trial and make recommendations to the TSC as to whether there are any ethical or safety issues that may necessitate a modification to the protocol or closure of the trial. We propose using the DAMOCLES charter for independent DMCs (IDMCs) as our reference point, which will be agreed in advance by the TSC. It is anticipated that both the TSC and the DMC would meet twice a year, once face-to-face and once via teleconferencing. The CI, all PIs, study coordinators, research nurses, and BRTC personnel will have undertaken the mandatory GCP training.

### Regulatory issues

#### Ethics approval

The CI obtained approval from the South Central – Oxford B Research Ethics Committee (14/SC/0237). The study must be submitted for Site Specific Assessment (SSA) at each participating NHS Trust. The CI will require a copy of the Trust Research and Development (R&D) approval letter before accepting participants into the study from that Trust (see Additional file 1 for a complete listing of supporting Trusts). The study will be conducted in accordance with the recommendations for physicians involved in research on human subjects adopted by the 18th World Medical Assembly, Helsinki 1964 and later revisions.

#### Sponsor

NBT will act as the Sponsor for this trial. Delegated responsibilities will be assigned to the NHS trusts taking part in this trial.

#### Funding

The National Institute for Health and Research, HTA programme are funding this study (project number 12/140/01).

### Publication policy

The main forms of dissemination will be through the academic press, HTA monograph, guidelines and workshops for clinical staff and by lay summaries on websites and other more accessible forms for patients. All participants will be offered a lay summary of the main findings of the study. This will be adapted for dissemination through public channels. Dissemination to clinicians will be through papers in major urology journals and conferences (e.g. the European Association of Urology), workshops and presentations to national meetings: e.g. the British Association of Urological Surgeons (BAUS), which is the specialist body with the responsibility for guiding clinical practice, policy matters, research priorities, governance and training in matters related to male LUTS.

Sub-studies will also be conducted on the trial results, written up and submitted for publication in peer-reviewed journals.

The success of the study depends entirely on the wholehearted collaboration of a large number of men undergoing investigation for BPO surgery, as well as their nurses and doctors. For this reason, chief credit for the study will be given, not to the committees or central organisers, but to all those who have collaborated in the study. The results of the study will be reported first to study collaborators. The main report will be drafted by the PMG and circulated to all clinical collaborators for comment. The final version will be agreed by the Steering Committee before submission to the funders (National Institute for Health Research (NIHR) HTA) and subsequent publication in a peer-reviewed journal, on behalf of all the UPSTREAM collaborators.

To safeguard the integrity of the main trial, reports of explanatory or satellite studies will not be submitted for publication without prior agreement from the PMG.

We intend to maintain interest in the study by publication of UPSTREAM newsletters at intervals for participants, staff and collaborators. Once the main report has been published, a lay summary of the findings will be sent in a final UPSTREAM newsletter to all involved in the trial.

## Discussion

The aim of this trial is to establish whether a care pathway not including invasive urodynamics is no worse than one in which it is included, in men who are considering further treatment where surgery might be an option for BOO. If successful, trial results could rationalise the diagnostic process and ensure its acceptability to patients and advocate whether urodynamics should be recommended or not in the future in relation to BOO; a conclusion the NICE Guideline Development Group indicated was pertinent and timely.

Here, we briefly discuss some of the key challenges we encountered during the first year of this trial, including strategic findings from our internal feasibility assessments, to provide valuable insight for research groups conducting future similar trials.

### Patient diagnostic care pathways

The second feasibility phase in particular, clearly showed that hospitals of any type (teaching or district general) receive sufficient inward referrals to expect delivery of projected recruitment. However, what came to light is that care pathways vary between each of the participating hospitals; more so than originally anticipated. All hospitals are structured differently and needed diverse approaches in terms of implementing successful identification of patients and recruitment strategies. Finding out where inward referrals to the urology department are triaged has proved helpful for screening patients, as is finding where medical notes go to after general clinics; this helps to avoid missing potentially eligible patients.

### Involvement of urologists as well as research nurses

Recruitment rates were enhanced greatly by including urologists (local PIs and research fellows) in the identification of patients rather than relying solely on research nurses. We observed that the more engaged the PI was with the trial, the smoother the initial recruitment phase. This included the PI helping the research nurses the first time they screen notes, regularly screening themselves and generally encouraging the other consultants and nurses and also involving the surrounding clinical care team. Once patients are identified, the large majority of men agree to participate, as the randomisation is between two standard NHS diagnostic pathways, at the end of which the patient chooses his treatment.

### Importance of site initiation visits and ongoing communication

We found that face-to-face site initiation visits, and follow-up visits, were crucial to getting recruitment started in each centre promptly and successfully. Many of the hospital research teams work on various studies simultaneously, so receiving direct training and encouragement helped to get them to keep pushing for UPSTREAM recruitment. Having a urology-trained lead research nurse dedicated solely to UPSTREAM has been invaluable; besides assisting with on-going communication and the monitoring of research centres, the dedicated lead research nurse provides a direct point of contact for nurses especially, to offer trouble-shooting, general support and encouragement.

Furthermore, sending out a monthly newsletter comparing sites’ recruitment figures and indicating monthly recruitment targets has also proved to be a useful tool to keep sites’ focus on recruitment. This created a positive element of competition amongst centres. Useful ‘hints and tips’ documents on recruitment strategies, and other relevant training materials, have also been well received.

Using multiple methods of communication (such as newsletters, emails, trial-specific website [46], Twitter and telephone calls) has proved essential to cater for a wide variety of preferences.

### Regular monitoring

Finally, it has been fundamental to the trial to ensure consistent monitoring of recruitment progress, continued understanding of the trial, and all operating procedures. Problems have been dealt with swiftly as a result and suitable modifications implemented.

## Trial status

Recruitment began in October 2014.

## Additional file

Additional file 1:
**Participating hospitals and supporting NHS Trusts that provided local research and development (R&D) approval.** (DOCX 15 kb)

## Abbreviations

AE: adverse event
AIS: Assessment Information Sheet
AUA: American Urological Association
BAUS: British Association of Urological Surgeons
BOO: bladder outlet obstruction
BPO: benign prostatic obstruction
BRTC: Bristol Randomised Trials Collaboration
CI: Chief Investigator
CISC: clean intermittent self-catheterisation
CG: clinical guideline
CONSORT: Consolidated Standards of Reporting Trials
CRF: case report forms
DMC: Data Monitoring Committee
EQ-5D-5 L: EuroQol Group’s 5 dimension health status questionnaire
GCP: Good Clinical Practice
GP: general practitioner
HES: Hospital Episodes Statistics
HRQOL: health-related quality of life
HTA: Health Technology Assessment
ICIQ-DUA: International Consultation on Incontinence Questionnaire – Detrusor Underactivity
ICIQ-MLUTS: International Consultation on Incontinence Questionnaire – Male Lower Urinary Tract Symptoms
ICIQ-MLUTSsex: International Consultation on Incontinence Questionnaire – sexual function associated with Male Lower Urinary Tract Symptoms
ICIQ-UDS-S: ICIQ urodynamics satisfaction
IDMC: independent data monitoring committee
IPSS: International Prostate Symptom Score
ISRCTN: International Standard Randomised Controlled Trial Number
ITT: intention-to-treat
IVR: interactive voice response
LUTS: lower urinary tract symptoms
NBT: North Bristol NHS Trust
NHS: National Health Service
NICE: National Institute for Health and Care Excellence
NIHR: National Institute for Health Research
OAB: overactive bladder syndrome
PI: Principal Investigator
PIS: Patient Information Sheet
PMG: Project Management Group
PP: patient panel
PPI: patient and public involvement
PSA: prostate specific antigen
PVR: post-void residual
QALY: quality-adjusted life years
Q_max_: maximum urinary flow rate
R&D: Research and Development
RCT: randomised controlled trial
RUL: Resource Use Log
SD: standard deviation
SAE: serious adverse event
SSA: Site Specific Assessment
TSC: Trial Steering Committee
TURP: transurethral resection of prostate
UK: United Kingdom
UDS: urodynamic studies
VV: voided volume
