# Supplementary material for: Urodynamics for Prostate Surgery Trial; Randomised Evaluation of Assessment Methods (UPSTREAM) for diagnosis and management of bladder outlet obstruction in men: study protocol for a randomised controlled trial
Source: Trials. 2015 Dec 10;16:567. doi: 10.1186/s13063-015-1087-1 (PMC4676182; doi:10.1186/s13063-015-1087-1)
Supplement: Additional file 1: — Participating hospitals and supporting NHS Trusts that provided local research and development (R&D) approval. (DOCX 15 kb) [file 13063_2015_1087_MOESM1_ESM.docx]

Additional File 1: Participating Hospitals and supporting NHS Trusts that provided local research and development (R&D) approval.

| **Hospital Name** *(in order of opening)* | **R&D Approvals Supporting NHS Trust** |
| --- | --- |
| 1. Southmead Hospital, Bristol | North Bristol NHS Trust |
| 1. Freeman Hospital, Newcastle upon Tyne | The Newcastle upon Tyne Hospitals NHS Foundation Trust |
| 1. Royal Devon and Exeter Hospital, Exeter | Royal Devon and Exeter NHS Foundation Trust |
| 1. Musgrove Park Hospital, Taunton | Taunton and Somerset NHS Foundation Trust |
| 1. Southport and Formby District General Hospital, Southport | Southport and Ormskirk Hospital NHS Trust |
| 1. Kingston Hospital, Kingston upon Thames | Kingston Hospital NHS Foundation Trust |
| 1. Royal Hallamshire Hospital, Sheffield | Sheffield Teaching Hospitals NHS Foundation Trust |
| 1. Epsom General Hospital, Epsom | Epsom and St Helier University Hospitals NHS Trust |
| 1. Queen Elizabeth Hospital, Birmingham | University Hospitals Birmingham NHS Foundation Trust |
| 1. Kent and Canterbury Hospital, East Kent and Canterbury | East Kent Hospitals NHS Foundation Trust |
| 1. Salisbury District General Hospital, Salisbury | Salisbury NHS Foundation Trust |
| 1. Lister Hospital, Stevenage | East and North Hertfordshire NHS Trust |
| 1. Churchill Hospital, Oxford | Oxford University Hospitals NHS Trust |
| 1. The James Cook University Hospital, Middlesbrough | South Tees Hospitals NHS Foundation Trust |
| 1. The Queen Elizabeth Hospital, King’s Lynn | The Queen Elizabeth Hospital King’s Lynn NHS Foundation Trust |
| 1. Royal Free Hospital, London | Royal Free Hospital London NHS Foundation Trust |
| 1. Royal Liverpool University, and Broadgreen, Hospitals | The Royal Liverpool and Broadgreen University Hospitals NHS Trust |
| 1. Torbay Hospital, Torbay | Torbay and South Devon NHS Foundation Trust |
| 1. Southampton General Hospital, Southampton | University Hospital Southampton NHS Foundation Trust |
| 1. Kettering General Hospital, Kettering | Kettering General Hospital NHS Foundation Trust |
| 1. Charing Cross Hospital, London | Imperial College Healthcare NHS Trust |
| 1. Royal Berkshire Hospital, Reading | Royal Berkshire NHS Foundation Trust |
| 1. Derriford Hospital, Plymouth | Plymouth Hospitals NHS Trust |
| 1. West Cumberland Hospital, Cumbria | North Cumbria University Hospitals NHS Trust |
| 1. Sunderland Royal Hospitals, Sunderland | City Hospitals Sunderland NHS Foundation Trust |
| 1. St George’s Hospital, London | St George’s University Hospitals NHS Foundation Trust |
